# The Landscape of Immunotherapy in Advanced NSCLC: Driving Beyond PD-1/PD-L1 Inhibitors (CTLA-4, LAG3, IDO, OX40, TIGIT, Vaccines)

**DOI:** 10.1007/s11912-021-01124-9

**Published:** 2021-08-27

**Authors:** Andrea De Giglio, Alessandro Di Federico, Giacomo Nuvola, Chiara Deiana, Francesco Gelsomino

**Affiliations:** 1 Division of Medical Oncology, IRCCS Azienda Ospedaliero-Universitaria Di Bologna, Via Albertoni 15, Bologna, Italy; 2 Department of Experimental, Diagnostic and Specialty Medicine, Alma Mater Studiorum University of Bologna, Bologna, Italy

**Keywords:** Non-small cell lung cancer, Immunotherapy, CTLA4, LAG3, IDO, OX40, TIGIT, Vaccines

## Abstract

**Purpose of Review:**

In this review, we analyzed the current landscape of non-PD-(L)1 targeting immunotherapy.

**Recent Findings:**

The advent of immunotherapy has completely changed the standard approach toward advanced NSCLC. Inhibitors of the PD-1/PD-L1 axis have quickly taken place as first-line treatment for NSCLC patients without targetable “driver” mutations. However, a non-negligible portion of patients derive modest benefit from immune-checkpoint inhibitors, and valid second-line alternatives are lacking, pushing researchers to analyze other molecules and pathways as potentially viable targets in the struggle against NSCLC.

**Summary:**

Starting from the better characterized CTLA-4 inhibitors, we then critically collected the actual knowledge on NSCLC vaccines as well as on other emerging molecules, many of them in their early phase of testing, to provide to the reader a comprehensive overview of the state of the art of immunotherapy in NSCLC beyond PD-1/PD-L1 inhibitors.

## Introduction

In the last decade, the rise of immunotherapy in the fight against cancer has completely changed the therapeutic paradigms of several tumors. Non-small cell lung cancer (NSCLC) was one of the first cancers that saw its therapeutic approach shift from the previous standard of care toward immunotherapy.

The complexity of interactions between cancer and the immune system is summarized within the immune editing theory, which is a dynamic process of reciprocal balance between the host and the guest, composed of three phases: elimination, equilibrium, and escape [1].

In the elimination phase, aberrant cells’ development induces an efficacious innate and acquired response leading to tumor killing. Some tumor clones succeed in surviving the cytotoxic activity and enter into a phase of quiescence characterized by an absence of significant growth.

Interestingly, the constant pressure of the adaptive immune system acts as a natural selection, which fosters the sub-clones capable of deploying a strategy of subsistence characterized by successful replication and elusion of the immune response.

In particular, the programmed cell death 1 (PD-1) receptor elicits a negative co-stimulatory signal that leads to the T-cell receptor (TCR) down off, and it is, thus, physiologically involved in the mechanism of immune tolerance and limitation of the immune response [2]. Cancer cells upregulate the expression of the PD ligand 1 (PD-L1) in order to escape from acquired T cell recognition within the tumor microenvironment (TME).

Thus, several monoclonal antibodies (mAb) directed against the so-called immune checkpoints (ICI) have been developed and represent the cornerstone of the current care strategy for advanced NSCLC patients.

Firstly, single-agent ICIs (nivolumab, atezolizumab, pembrolizumab) overwhelmed the standard chemotherapy in the second-line setting, offering considerable benefits in terms of survival and safety [3–6].

Concerning the first-line, pembrolizumab unseated the platinum-based doublets for a selected population of patients harboring high expression of PD-L1 [7]. Recently, different combinations of chemotherapy and ICIs change the treatment landscape in the first-line setting regardless of PD-L1 status and histology [8]. Furthermore, the use of quadruple combination therapy of atezolizumab, bevacizumab, and platinum-based chemotherapy showed efficacy in a wider population, including oncogene-addicted NSCLC patients [9].

Unfortunately, long survivorship does not go past 19% at 3 years and 16% at 5 years, underlying that only a small portion of patients benefits from anti-PD-1/PD-L1 strategies [10]. Remarkably, a recent update of the Keynote 024 study showed that 31.9% of pembrolizumab-treated patients, as single-agent upfront strategy [7], were alive at 5 years [11].

**Fig. 1:**
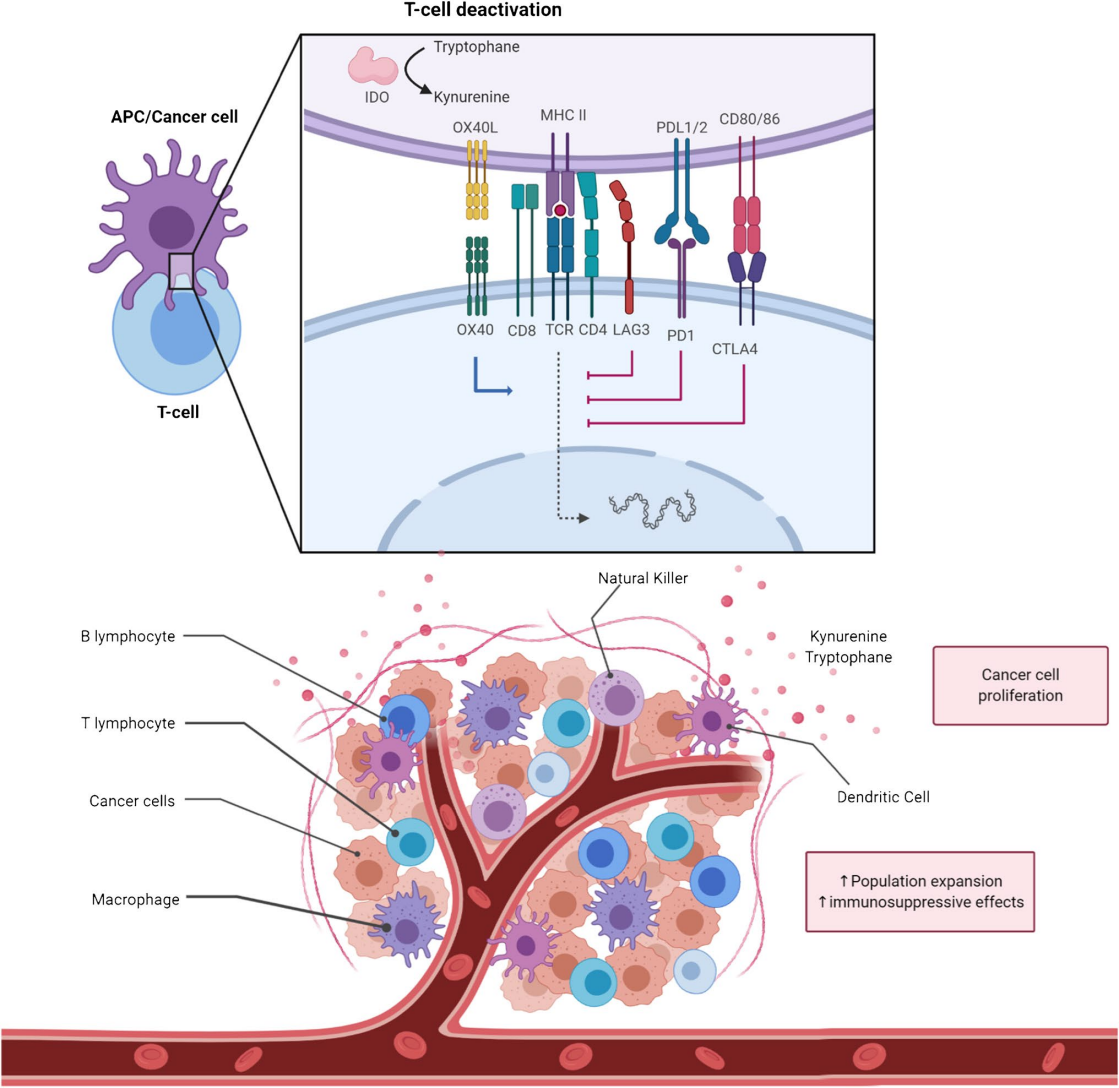
Interaction between T cell and APC/Cancer cell within the tumor microenvironment. APC: antigen-presenting cell; MHC: major histocompatibility complex; CTLA-4: cytotoxic T-lymphocyte antigen 4; LAG3: lymphocyte activation gene-3; IDO: indoleamine 2,3-dioxygenase 1; PD(L)1: programmed death (ligand) 1. Created with BioRender.com

The resistance to the inhibition of the PD-1/PD-L1 axis can either be primary or acquired during treatment. Several features have been associated with resistance to ICIs in the case of “immune hot” tumors, ranging from the activation of alternative negative co-stimulatory pathways to the polarization toward an immunosuppressive microenvironment with the interplay of innate or acquired immune cells [12] (Fig. 1). Conversely, the condition of “immune exclusion” is characterized by the scarce ability of immune cells to penetrate the core of the tumor due to peripheral inhibition or total absence of cytotoxic T-cells, leading to the so-called immune desert [13, 14].

In light of these data, different therapeutic strategies were tested to find novel predictive biomarkers and alternative or synergistic treatments besides PD-(L)1 inhibitors.

The growing knowledge of the immune-escape mechanisms fostered the development of different immunotherapeutic agents.

In this review, we collected the current evidence on immunotherapeutic agents beyond PD-1/PD-L1 inhibitors in advanced NSCLC. Starting from a specific biological background, we critically looked at the data yet available and ongoing trials to provide a comprehensive overview of the current state of immunotherapy for advanced NSCLC.

## CTLA-4

The cytotoxic T lymphocyte antigen 4 (CTLA-4) is an immunoglobulin superfamily member receptor mainly expressed on the surface of activated and regulatory T-cells that binds with high-affinity CD80 and CD86 on antigen-presenting cells (APC). The biological role of CTLA-4 is the inhibition of T-cell priming, activation, and migration. In particular, it acts as a negative feedback inducer for activated T-cells within the APC-T cell synapsis context [15]. Furthermore, regulatory T-cells constitutively express CTLA-4 and interact with APC, inducing the upregulation of indoleamine 2,3-dioxygenase (IDO), contributing to create an extrinsic immunosuppressive mechanism [16]. Given that cancer cells do not exhibit CD80 or CD86, the antineoplastic role of anti-CTLA4 antibodies is mainly connected with T-cell priming within both lymph nodes and TME. Thus, this checkpoint acts in a different phase than the PD-1/PD-L1 axis, and targeting both pathways represents a potentially effective combination to overcome the resistance to ICI monotherapy [17, 18]. Ipilimumab is a fully human IgG-1 mAb directed against CTLA-4 that showed promising results when combined with nivolumab in metastatic melanoma [19], opening the way for its use in other malignancies, including advanced NSCLC. The open-label, phase I, CheckMate-012 trial investigated the safety of nivolumab and ipilimumab combination on a cohort of chemotherapy-naïve advanced NSCLC patients, assessing a tolerable safety profile [20].

The phase II Checkmate-568 trial recruited treatment-naive advanced NSCLC patients, testing the combination of nivolumab 3 mg/kg every 2 weeks plus ipilimumab 1 mg/kg every 6 weeks. ORR, which was the primary endpoint, was 30% in the overall study population. PD-L1 and the tumor mutational burden (TMB) resulted in being independent predicting factors for the combination treatment efficacy. The ORR and median progression-free survival (PFS) were higher for the subgroup of patients harboring PD-L1 ≥ 1% than those with < 1% expression, as ORR was 41% versus 15% and median PFS was 6.8 months versus 2.8 months, respectively. Notably, the subgroup expressing a TMB of 10 or more mutations per megabase (mut/mb) had improved ORR (44% vs. 12%) and median PFS (7.1 months vs. 2.6 months). Grade 3 and 4 AEs occurred in 29% of patients, mostly skin and gastrointestinal events (3 and 5%), and increased lipase (6%) [21].

These findings led to the development of a subsequent randomized, open-label, first-line, phase III trial named CheckMate-227 [22]. Advanced NSCLC patients were randomly assigned to receive nivolumab plus ipilimumab, nivolumab alone (only for PD-L1 > 1%), nivolumab plus chemotherapy (only for PD-L1 < 1%), or chemotherapy alone. The trial was pre-planned to assess the efficacy according to PD-L1 expression. In the group with a PD-L1 expression level of 1% or more, the median overall survival (OS) was 17.1 months with nivolumab plus ipilimumab and 14.9 months with chemotherapy, reaching a 2-year OS rate of 40.0% and 32.8%, respectively. An OS benefit was also observed in patients with PD-L1 < 1%, with a median of 17.2 months with nivolumab plus ipilimumab and 12.2 months with chemotherapy. In the overall population, regardless of PD-L1 status, the median OS was 17.1 months with nivolumab plus ipilimumab and 13.9 months with chemotherapy. No additional OS benefit with the combination therapy was documented in patients with TMB > 10 mut/mb. 32.8% of patients treated with nivolumab plus ipilimumab and 36.0% of those treated with chemotherapy experienced grade 3–4 treatment-related AEs. As previously described, the most frequent irAEs were cutaneous and endocrine [22]. The association between nivolumab and ipilimumab was recently explored in addition to two cycles of chemotherapy, as compared to four cycles of chemotherapy, among 719 patients with previously untreated advanced NSCLC [23]. The phase III CheckMate 9-LA trial documented improved outcomes in the experimental arm, either in terms of PFS (6.7 months vs 5 months), OS (15.6 months vs 10.9 months), or ORR (38.2% vs 24.9%). However, the risk of grade ≥ 3 TRAEs was increased with the combination strategy (47% vs 38%, respectively). Moreover, ipilimumab was tested in addition to pembrolizumab aiming to enhance its effectiveness in the population of untreated NSCLC patients with high PD-L1 expression (≥ 50%) [24]. However, the phase III Keynote 598 trial reported the absence of increased benefit with the addition of the anti-CTLA-4 agent compared to pembrolizumab alone. Median PFS and OS were similar in both arms, but a significantly increased risk of grade ≥ 3 TRAEs was reported in the experimental arm (35.1% vs 19.6%, respectively).

Tremelimumab is a fully human, anti-CTLA-4, IgG2 mAb. In combination with the anti-PD-L1 agent durvalumab, tremelimumab was preliminarily studied to treat advanced NSCLC in a phase Ib trial. The combination cohort who received durvalumab 20 mg/kg every 4 weeks and tremelimumab 1 mg/kg showed an acceptable toxicity profile, with grade 3–4 events in 30% of the patients, mostly gastrointestinal, and the antitumor activity was irrespective of PD-L1 status [25].

These findings led to the phase III MYSTIC trial, which tested durvalumab alone or in combination with tremelimumab as an upfront strategy in advanced NSCLC patients. Median OS and PFS did not significantly differ from standard first-line chemotherapy. Nonetheless, a median OS improvement in the durvalumab monotherapy arm was documented among patients with PD-L1 expression ≥ 25% compared to chemotherapy (16.3 versus 12.9 months). Similarly, better OS for the immuno-combination compared to chemotherapy (16.5 versus 10.5 months) was demonstrated in patients with TMB ≥ 20 mut/mb. Treatment-related AEs of grade ≥ 3 occurred in 22.9% of patients treated with durvalumab plus tremelimumab, with 10.8% of them immune-related, most frequently gastrointestinal and pulmonary [26]. The combination of durvalumab and tremelimumab was furtherly investigated in the phase III NEPTUNE trial, where patients with untreated IV stage NSCLC were randomized to receive durvalumab plus tremelimumab or standard platinum-based doublet. The primary analysis in patients with high TMB (≥ 20 mut/mb) did not meet the primary endpoint of reducing mortality [27]. To date, the ongoing POSEIDON trial is testing the combination of durvalumab plus chemotherapy or plus tremelimumab and chemotherapy versus chemotherapy alone as first-line therapy in advanced NSCLC patients. A press release reported that both experimental arms met the primary endpoint, as improved PFS was documented, even if official results are not available so far. At the moment of publication, OS data were not mature yet [27].

To date, several trials involving different PD-(L)1 inhibitors associated with anti-CTLA 4 drugs are ongoing in first and further lines for advanced NSCLC patients (Table 1).

**Table 1:** Ongoing trials evaluating novel immunotherapeutic strategies beyond PD-(L)1 axis inhibition

Target	Drug combination	Setting	Phase	Primary outcome	No pts ext	clinicaltrial.gov identifier
CTLA-4	Nivolumab + ipilimumab + nintedanib	Locally advanced or metastatic NSCLC	I/II	Safety, tolerability, ORR	98	NCT03377023
	Nivolumab + NKTR-214 + / − ipilimumab	Locally advanced or metastatic NSCLC	I/II	Safety, tolerability, ORR	n.a	NCT02983045
	- Nivolumab + / − ipilimumab + hypofraxionated RT on bone lesion—nivolumab + / − ipilimumab + hypofraxionated RT on metastatic lesion	Locally advanced or metastatic NSCLC eligible for palliative RT on a metastatic lesion	I	Safety	24	NCT03509584
	Nivolumab + ipilimumab	Stage IV or recurrent, treatment-naive NSCLC	II	ORR	250	NCT03001882
	Nivolumab + ipilimumab + paclitaxel	Stage IV treatment-naive NSCLC	II	PFS	49	NCT03573947
	Nivolumab + ipilimumab + cisplatin/carboplatin and pemetrexed or paclitaxel	Stage IV treatment-naive NSCLC	III	OS	700	NCT03215706
	1) 6 months nivolumab + ipilimumab followed by observation and nivolumab + ipilimumab in case of progression 2) Nivolumab + ipilimumab	Stage IV treatment-naive NSCLC	III	PFS	1360	NCT03469960
	SBRT followed by durvalumab + tremelimumab	Stage IV NSCLC with 6 or less extra cranial sites for SBRT	Ib	Safety and tolerability	21	NCT03275597
	1) Durvalumab + tremelimumab 2) 4 cycles of cisplatin/carboplatin + pemetrexed or gemcitabine + durvalumab + tremelimumab followed by maintenance durvalumab + / − pemetrexed	Stage IV treatment-naive NSCLC	II	OS	301	NCT03057106
	1) Durvalumab + tremelimumab + chemotherapy 2) Durvalumab + chemotherapy 3) Chemotherapy	Stage IV treatment-naive NSCLC	III	PFS and OS	1000	NCT03164616
	Pembrolizumab + / − ipilimumab	Stage IV treatment-naive NSCLC, PD-L1 â‰¥ 50%	III	PFS and OS	548	NCT03302234
	1) Cemiplimab + ipilimumab 2) Cemiplimab + ipilimumab + chemotherapy	Stage IV or recurrent, treatment-naive NSCLC, PD-L1 â‰¥ 50%	III	PFS		NCT03515629
LAG3	Neoadjuvant nivolumab + / − relatlimab	Stage I, II, and IIIa NSCLC	II	Feasibility	60	NCT04205552
	Pembrolizumab + IMP321	Locally advanced or metastatic NSCLC, treatment-naive or PD-x refractory	II	ORR		NCT03625323
IDO	1) Pembrolizumab + epacadostat + platinum-based chemotherapy 2) Pembrolizumab + platinum-based chemotherapy	Stage IV treatment-naive NSCLC	II	ORR	233	NCT03322566
	1) Pembrolizumab + epacadostat 2) Pembrolizumab	Stage IV treatment-naive NSCLC, PD-L1 â‰¥ 50%	II	ORR	154	NCT03322540
	Indoximod + docetaxel + tergenpumatucel-L	Stage IV pretreated NSCLC	I	Safety, PFS	16	NCT02460367
CD137	Nivolumab + intratumoral urelumab	Advanced solid tumors (including NSCLC)	I/II	Safety, RR	32	NCT03792724
	Urelumab	Advanced solid tumors (including NSCLC)	I	RR, DLTs	18	NCT02534506
	Avelumab + other immunotherapy agents (including utomilumab)	Advanced solid tumors (including NSCLC)	Ib/II	RR, DLTs	620	NCT02554812
OX40	INBRX-106 + / − pembrolizumab	Advanced solid tumors (including NSCLC)	I	Safety, MTD, and/or RP2D	150	NCT04198766
	GSK3174998 + / − pembrolizumab	Advanced solid tumors (no more than 5 lines of therapy received)	I	Safety, DLT, activity	142	NCT02528357
	MOXR0916 + atezolizumab	Advanced solid tumors (including NSCLC)	I	DLT, safety	610	NCT02410512
	MEDI6383 + / − MEDI4736/durvalumab	Advanced solid tumors including NSCLC (no more than 5 lines of therapy received)	I	Safety	39	NCT02221960
	IBI101 + / − sintilimab	Advanced solid tumors (including NSCLC)	I	DLT, safety	80	NCT03758001
	GB-A445 + / − tislelizumab	Advanced solid tumors (including NSCLC)	I	Safety, DLT, MTD, RP2D, ORR	68	NCT04215978
	MEDI0562	Advanced solid tumors including NSCLC (no more than 3 lines of therapy received)	I	DLT, safety	56	NCT02318394
	MEDI0562 + tremelimumab, MEDI0562 + durvalumab	Advanced solid tumors including NSCLC (no more than 3 lines of therapy received)	I	DLT, safety	58	NCT02705482
	BMS-986178 + / − ipilimumab + / − nivolumab + / − DPV-001 vaccine/cyclophosphamide	Advanced solid tumors (including NSCLC), second or subsequent line	I/IIa	Safety, lab test abnormalities	207	NCT02737475
	INCAGN01949	Advanced solid tumors (including NSCLC)	I/II	Safety	87	NCT02923349
	INCAGN01949 + nivolumab, + / − ipilimumab	Advanced solid tumors (including NSCLC)	I/II	Safety, ORR	52	NCT03241173
	PF-04518600 + / − utomilumab	Advanced solid tumors (including NSCLC in Part B of the trail), treated with anti-PD-L1/PD-1 therapy	I	DLT, safety	176	NCT02315066
TIGT	Tiragolumab + atezolizumab	Chemotherapy-naïve patients with advanced non-small cell lung cancer	II	ORR, PFS	135	NCT03563716
	Tiragolumab + atezolizumab	Previously untreated locally advanced unresectable or metastatic PD-L1-selected non-small cell lung cancer	III	PFS, OS	500	NCT04294810
	AB154 + / − AB122 (zimberelimab)	Advanced solid tumors (including NSCLC	I	Safety	66	NCT03628677
	AB154 + zimberelimab, AB154 + zimberelimab + AB928	D-L1 positive, advanced non-small cell lung cancer	II	ORR, PFS	150	NCT04262856
	Vibostolimab (MK-7684) + / − pembrolizumab or + pembrolizumab + pemetrexed + carboplatin	Advanced solid tumors (including NSCLC)	I	DLT, safety	432	NCT02964013
	Pembrolizumab + MK-7684, + carboplatin-paclitaxel or + pemetrexed	Treatment-naïve participants with advanced NSCLC	II	ORR, PFS	90	NCT04165070
	BMS-986207 + / − nivolumab	Advanced solid tumors (including NSCLC)	I/IIa	Safety, lab test abnormalities	170	NCT02913313
	ASP8374 + / − pembrolizumab	Advanced solid tumors (including NSCLC)	Ib	DLT, safety, pharmacokinetics	363	NCT03260322

*PD-(L)1* programmed death (ligand) 1, *ORR* overall response rate, *PFS* progression-free survival, *OS* overall survival, *RR* response rate, *DLT* dose-limiting toxicity, *MTD* maximum tolerated dose, *RP2D* recommended phase 2 dose

## LAG3

The lymphocyte activation gene-3 (LAG3; CD223) is an immune inhibitory receptor, expressed on activated T-cells, natural killer cells (NK), and B-cells, which binds the major histocompatibility complex (MHC) of class II. Some of the main functions of LAG3 include the inhibition of Th1-cell proliferation and the reduction of IL-2, IFN- γ, and TNF production [28].

LAG-3 also binds the liver sinusoidal endothelial cell lectin (LSECtin), a dendritic cell-specific intercellular adhesion molecule-3-grabbing non-integrin (DC-SIGN) family. A study on melanoma cells showed that this connection promotes the immune escape of cancer cells, inhibiting T-cells antitumor response [29].

Moreover, LAG3 is expressed on tumor-infiltrating lymphocytes (TILs) and takes part in the immune-escape mechanism. Although its mechanism of action has not been fully clarified, suppressive T-cells expressing LAG3 have a documented enhanced activity, whereas cytotoxic T-cells expressing LAG3 exhibit lower proliferation rates and decreased production of cytokines. As a result, the persistent upregulation of LAG3 leads to the exhaustion of the immune response.

These biological functions make LAG3 an interesting target for immunotherapy, especially in combination with other ICIs [30, 31].

Ieramilimab (LAG525) is a novel anti-LAG3 agent currently investigated within two trials, as monotherapy or in combination with PDR001, an experimental anti-PD-1 agent.

A phase I/II trial tested the safety and antitumor activity of LAG525 as single-agent or combined with PDR001 in patients with solid tumors, including NSCLC. The combination showed an acceptable safety profile and led to durable responses in 12 patients. Intriguingly, tumor re-biopsies showed a trend of conversion from immune-cold to immune-activated TME [32].

Ieramilimab alone or in combination with PDR001 was further studied in another phase I/II trial enrolling patients with different pretreated advanced malignancies. The combination showed promising activity in small cell lung cancer (SCLC), neuroendocrine tumors (NET), and diffuse large B-cell lymphoma. To date, no patients with advanced NSCLC have been enrolled (NCT03365791).

Relatlimab (BMS-986016) is another investigational anti-LAG3 agent initially tested in combination with nivolumab in a phase I/II trial enrolling patients with advanced melanoma who received prior immunotherapy. The combination showed an ORR of 11.5% and a DCR of 49%, with grade ≥ 3 AEs occurring in 10% of patients, mostly gastrointestinal. Remarkably, patients with LAG3 expression > 1% were more likely to achieve objective responses [33].

The combination of relatlimab and nivolumab is currently under investigation as neoadjuvant therapy for early-stage NSCLC (Table 1). To our knowledge, the combination of pembrolizumab with IMP321, a Soluble LAG-3 Fusion Protein, is the unique phase II ongoing study among a population of untreated, unresectable, or metastatic NSCLC patients (Table 1) [34].

## IDO1

Indoleamine 2,3-dioxygenase 1 (IDO1) is one of the three intracellular enzymes (IDO1, IDO2, and TDO) that acts as a critical step in the degradation of the amino acid tryptophan to kynurenine [35]. An in vitro study demonstrated that the administration of tryptophan analogs prevented allogeneic fetal rejection [36]. Subsequently, several studies showed that IDO1 plays an immunosuppressive role favoring the tumor immune escape [37].

In the context of TME, both tumor and immune cells, including stromal cells, lymphocytic cells, and dendritic cells, express IDO1. Also, IFN-γ, TNF-α, TGF-β, and other pro-inflammatory signals induce IDO1 cytosolic expression. The decrease of tryptophan and the increased levels of its metabolites, mediated by IDO1, lead to anergy and apoptosis of effector T-cells and the activation of regulatory T-cells [38]. Moreover, IDO1 seems to promote the inflammatory neovascularization of the tumor site, acting against the anti-angiogenic effect of IFN-γ [39].

Epacadostat, navoximod, and BMS-986205 directly inhibit IDO1, while indoximod is a tryptophan-mimicking agent that blocks mTORC1, a downstream protein complex acting as an immunosuppressive agent for T-cells in situations of cell stress and tryptophan deficiency [40].

Epacadostat is an oral molecule that selectively inhibits IDO1. In preclinical models, it increased the proliferation of effector T-cells and natural killer cells, reducing regulatory T-cells’ activation, especially when combined with other ICIs [41]. Phase I and II studies showed good tolerability and activity in several advanced solid tumors [42, 43].

Results from phase I/II of the ECHO-202/KEYNOTE-037 trial showed that the combination of epacadostat and pembrolizumab had good tolerability and antitumor activity in various solid tumors, including pretreated advanced NSCLC (ORR 35%). Most common grade 3–4 AEs were increased lipase (16%), fatigue, and rash (3%) [44].

Other combinations, such as epacadostat plus nivolumab and GDC-0919 plus atezolizumab, demonstrated limited efficacy in NSCLC patients [45, 46].

The enthusiasm around epacadostat fell after the failure of the phase III ECHO 301/KEYNOTE 252 trial where the combination of epacadostat with pembrolizumab was found not superior to pembrolizumab alone in advanced melanoma [47].

Despite disappointing these results, two phase III trials are currently evaluating epacadostat as first-line therapy in metastatic NSCLC (Table 1). One trial is investigating the combination of epacadostat and pembrolizumab in metastatic NSCLC with high expression of PD-L1 (NCT03322540).

Interestingly, another trial is investigating epacadostat in combination with pembrolizumab and platinum-based chemotherapy as first-line therapy (NCT03322566).

Several studies are ongoing testing the safety and efficacy of indoximod to treat various advanced solid malignancies. To date, indoximod in pretreated advanced lung cancer is currently tested in phase I trial of combination with docetaxel and in tergenpumatucel-L immunotherapy (NCT02460367).

The activity of navoximod, another investigational IDO1 inhibitor, was studied in a phase I clinical trial in association with atezolizumab in several advanced cancer, including NSCLC. The combination showed good tolerability and activity, but there was no evidence of benefit regarding the addiction of navoximod to anti-PD-L1 agent [48].

### CD137(4-1BB)

CD137 (also known as 4-1BB and TNFRSF9) is a co-stimulatory surface molecule belonging to the tumor necrosis factor receptor superfamily (TNFRS) [49], which includes a large number of proteins involved in cell proliferation, differentiation, and programmed cell death [50]. CD137 is expressed by several immune cells, including activated CD4 + and CD8 + lymphocytes and natural killer cells (NK) [51]. In mice, the interaction with its ligand, CD137L (4-1BBL), mostly expressed by antigen-presenting cells [52], results in the stimulation of T lymphocytes via the nuclear factor kappa-light-chain-enhancer of activated B cells (NF-κB), Jun amino-terminal kinases/stress-activated protein kinases (JNK/SAPK), and p38 mitogen-activated protein kinases (p38 MAPK) pathways [53]. CD137/CD137L signaling also affects cells expressing CD137L, through a process called reverse signal transduction [54]. Hence, this interaction also affects the innate immune response, controlling monocyte proliferation, survival, and maturation into Th1-inducing dendritic cells (DCs) [55, 56]. Moreover, CD137 may also decrease tumor-infiltrating regulatory T-cells [57]. The first evidence of enhanced antitumor immune response targeting CD137 with mAb dates to 1997, when Melero et al. demonstrated the eradication of large established tumors in mice through an elicited cytolytic T-cell activity [58]. In NSCLC, CD137L showed a positive correlation with early-stage, well-differentiated tumors and better OS [59, 60].

Urelumab (BMS-663513, clone 10C7; Bristol-Myers Squibb) is a CD137 agonist, non-ligand-blocking, fully human IgG4 mAb [61]. The phase I/II study presented in 2008 by Sznol et al. showed promising antitumor potential, although severe dose-dependent liver toxicities and hepatotoxicity-related deaths were reported [62]. Urelumab was then tested alone and associated with the PD-1 inhibitor nivolumab in patients with different tumors, including NSCLC [63]. No objective responses were obtained in the urelumab monotherapy arm, whereas only one of the 34 patients with NSCLC in the combination arm responded. The patient was naïve to PD-1/PD-L1 treatment, and his tumor expressed high levels of PD-L1. The disease control rate for NSCLC patients was 29% (21% for those who progressed on prior PD-1/PD-L1 treatment and 35% for PD-1/PD-L1 naïve patients, respectively). To date, two ongoing trials are evaluating urelumab in NSCLC patients (Table 1).

Utomilumab (PF-05082566, Pfizer) is a humanized 4-1BB agonist IgG2 mAb with high affinity and specificity [64]. Unlike urelumab, clinical data from the phase I study showed promising safety, as no significant transaminitis or other dose-limiting toxicities emerged in a cohort of 55 patients with advanced solid tumors [65]. ORR was 3.8%, while median PFS and OS were 1.7 and 11.2 months, respectively. Utomilumab was then tested in association with the PD-1 inhibitor pembrolizumab in 23 patients with advanced solid tumors [66]. ORR was 26%, including 1 out of 6 NSCLC patients who achieved a partial response (PR), and no treatment-related discontinuations were reported. Recently, the combination of utomilumab and mogamulizumab, a humanized mAb targeting C–C chemokine receptor 4 (CCR4, CD194) to deplete the CCR4 + T-reg lymphocyte population, was tested in patients with PD-1/PD-L1 refractory or relapsed tumors [67]. No dose-limiting toxicities occurred, and ORR was 4.2%. Of 10 patients with NSCLC, 1 achieved PR with a duration of response (DoR) of 2 months. Ongoing trials evaluating the efficacy and safety of utomilumab are resumed in Table 1.

## OX40

OX40, also known as CD134 or TNFRSF4, belongs to the TNFR family. This molecule provides co-stimulatory molecular signals in the late activation and survival phase of activated T-cells [68]. Its ligand, OX40L (or CD252), is mainly expressed by APCs, activated B-cells, macrophages, and NK cells [68].

The result of OX40 and OX40L interaction varies according to the type of cell expressing OX40. Its activation on CD4 + T-cells can enhance Th-1 immune response, promotion, and maintenance of the Th-2 subset or differentiation of CD4 + T-cells into the pro-inflammatory Th-9-lymphocyte subset but also contributing the maintenance of follicular helper T-cell functions [68]. Furthermore, T-reg cells are negatively regulated by the OX40 receptor, thus blocking the inhibitory activity of T-reg on CD4 + T cells [68]. In CD8 + cells, OX40 demonstrated a role in proliferation and antitumor activity, and it also promotes cooperation between CD4 + and CD8 + in tumor-suppressing functions [69].

Therefore, OX40-OX40L interaction has a robust immunological effect that can potentially be effective in cancer immunotherapeutic strategies. In immunogenic tumors, such as certain types of murine sarcoma, breast cancer, and colon cancer, the results of OX40 targeted therapy were promising [70]. On the other hand, in poorly immunogenic tumors, OX40 agonists may not be enough to provide the proper stimulus for the immune system; thus, the combination with different types of immunotherapy drugs has been proposed [68, 70]. Indeed, the use of OX40 agonists with ICIs, cytokines such as IL2 and IL12, chemotherapeutic drugs such as cyclophosphamide, or radiation therapy, has shown a synergic effect on different types of murine cancer[68, 71, 72].

At the time of this review, several OX40 agonists are under evaluation in clinical trials, all of them in the advanced/metastatic setting (Table 1).

MOXR0916 is an OX40 agonist mAb being tested alongside atezolizumab in solid malignancies. Although no official results have been announced yet, no treatment-related discontinuations had occurred in the dose-escalation cohorts according to a preliminary report, and no adverse effects leading to treatment discontinuation had occurred [73]. Further OX40 agonists being tested alone or combined with PD-(L)1 inhibitors are MEDI6383 alongside durvalumab, IBI101 with the anti-PD-1 drug sintilimab, and BGB-A445 in combination with the anti-PD-1 agent tislelizumab (NCT02221960) (NCT03758001) (NCT04215978).

Several trials are testing the triple combination of OX40 agonists, PD-1 inhibitors, and CTLA-4 inhibitors as well. A dose-escalation phase I trial is evaluating MEDI0562, an OX40 agonist, either as a monotherapy or in combination with the anti-CTLA-4 tremelimumab and the anti-PD-1 durvalumab (NCT02705482). Preliminary results showed that the novel agent was generally well tolerated, with 16% of patients experiencing grade 3 AEs (fatigue being the most common), while no grade 4 AE was reported [74].

Wang et al. evaluated BMS-986178, another OX40 agonist mAb, administered alone or in combination with nivolumab and/or ipilimumab in a phase I/II trial. The trial is still recruiting, but a preliminary analysis of the combination arm’s pharmacokinetics and pharmacodynamics showed efficient target engagement and an increase in peripheral T-cells activation [75].

Further trials evaluating the combination of OX40 agonists with PD-(L)1 and CTLA-4 inhibitors are described in Table 1.

OX40 agonists are also being tested with other types of immunotherapeutic agent, including the Toll-like receptor 9 (TLR9) agonist SD-101(NCT03831295), the 4-1BB agonist utomilumab (NCT02315066), and avelumab (NCT02554812) (NCT03217747).

## TIGIT

TIGIT (T-cell immunoreceptor with Ig and ITIM domains) gene encodes for an inhibitory immune receptor of the poliovirus receptor (PVR) family of immunoglobulin proteins [76, 77]. This receptor can be expressed on activated NK cells, CD4 + and CD8 + T-cells, and T-reg cells [78, 79]. TIGIT can bind three ligands: CD155 (also known as PVR), CD112 (Nectin-2 or PVRL2), and CD113 (PVRL3). Both CD155 and CD122 can be expressed by myeloid cells but can also be overexpressed in tumor cells, while TIGIT expression is often upregulated in TILs [78, 80, 81].

TIGIT shares its ligand, CD155, with the immune activator receptor CD226 (DNAM-1) and the receptor CD96 [78, 81]. TIGIT, DNAM-1, and CD96 are NECL proteins (receptors for nectin and nectin-like) expressed on T-cells and NK cells, and they have different affinities for CD155 and opposite functions, creating a complex mechanism that regulates the immune response [78, 82].

When TIGIT binds with CD155, a downregulation of T cells and NK functions ensues, but the exact mechanism of action underlying this interaction is still under debate. TIGIT seems to have an indirect role (helping to steer dendritic cells toward the production of inhibitory cytokines and inducing a phenotypes shift in macrophage toward the M2 anti-inflammatory profile) and a direct, cell-intrinsic inhibitory function that can manifest in several ways [78, 83]. One such mechanism is a consequence of the higher affinity of CD155 for the inhibitory TIGIT compared to the activator DNAM-1 [80, 84–86].

Besides preventing DNAM-1 signaling, TIGIT can also influence inhibitory pathways through its cytoplasmic tail via the inhibition of phosphoinositide 3- kinase (PI3K) and mitogen-activated protein kinase (MAPK) signaling cascade, causing the downregulation of NK cells killing functions. Moreover, it can also impair nuclear factor kappa B (NF-κB) activation, halting IFN-γ production, and can lead to a decreased expression of the T-cell receptor (TCR) and other molecules involved in the TCR/CD28 signaling [86–88]

Finally, TIGIT can be found highly expressed by certain subsets of T-regs which, compared to TIGIT-T-regs, perform a more efficient T-cell suppression [89].

As in many types of tumors, increased TIGIT expression in TILs can be found in NSCLC. Of note, its overexpression can correlate with increased levels of other immune inhibitory receptors (such as PD-1, LAG-3, TIM-2) and lower levels of activator receptors (like DNAM) [80].

Following these observations, several studies tested antagonistic mAbs targeting TIGIT to increase cancer immune response. In preclinical mouse models, the use of single-agent anti-TIGIT mAbs was often insufficient in causing a significant response in subcutaneous tumors. Thus, a double agent therapy was implemented, consisting of TIGIT targeting agents and PD-1 inhibitors, obtaining better results [80].

Etigilimab (OMP-313M32) is an anti-TIGIT mAb used as a single-agent or in combination with nivolumab in a phase I trial among several advanced solid malignancies. Despite promising results documented in the phase Ia trial for etigilimab, both in terms of safety and antitumor activity, the phase Ib was not carried on due to sponsor decision (NCT03119428).

In 2018, a phase II study (NCT03563716), evaluating the safety and efficacy of the anti-TIGIT mAb tiragolumab (MTIG7192A) plus atezolizumab in chemotherapy-naive patients with advanced PD-L1-selected NSCLC, was started. The preliminary results of this trial showed that the combination arm of tiragolumab and atezolizumab improved ORR (37.3% vs. 20.6%) and PFS (5.6 vs. 3.9 months) compared to the atezolizumab-placebo arm, with a favorable safety profile. In January of 2021, these results lead to the FDA granting a Breakthrough Therapy Designation (BTD) for tiragolumab in combination with atezolizumab for the first-line treatment of patients with non oncogene-addicted, high PDL-1 expression, metastatic NSCLC [90].

Of note, the anti-TIGIT MK-7684 is also being studied combined with standard chemotherapy plus pembrolizumab (NCT02964013) (NCT04165070).

Other ongoing trials, testing anti-TIGIT antibodies alone or in combination with anti-PD(L)-1 molecules and other immunotherapeutics, can be found in Table 1.

## Vaccines

**Fig. 2:**
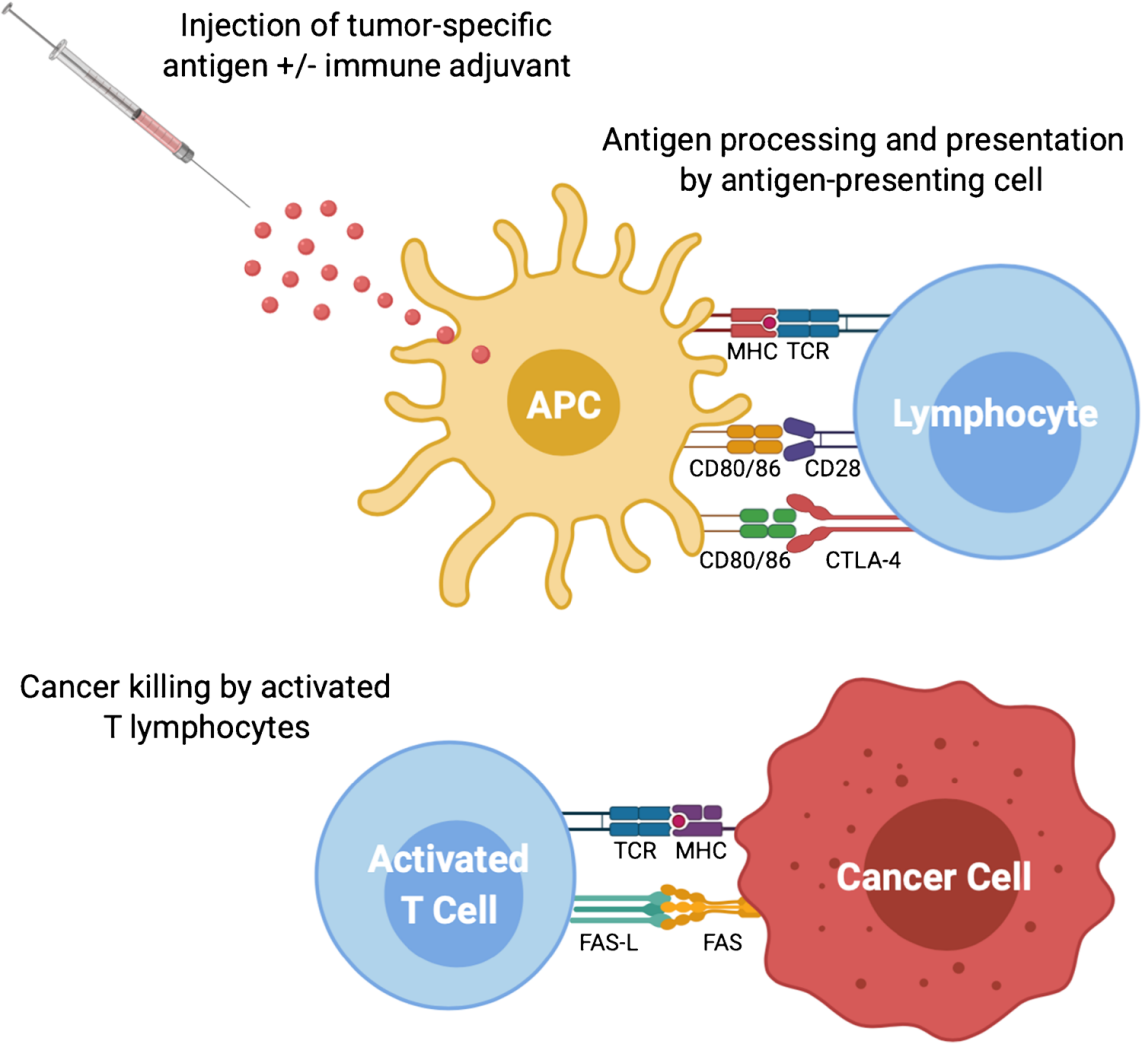
Tumor vaccines and anti-cancer immunity. MHC: major histocompatibility complex; TCR: T-cell receptor; CTLA-4: cytotoxic T-lymphocyte antigen 4. Created with BioRender.com

With the aim to enhance T-cells responses against specific tumor antigens, many NSCLC vaccines have been tested in large phase III trials during the last decade [91] (Fig. 2).

Belagenpumatucel-L is an allogeneic vaccine obtained transfecting NSCLC cell lines with a plasmid containing transforming growth factor β2 (TGF-β2) antisense transgene [92]. Two phase II trials with a total of 96 NSCLC patients documented promising safety and efficacy results [77, 92]. Nevertheless, the subsequent phase III trial testing belagenpumatucel-L versus placebo as maintenance therapy did not show difference in OS nor PFS [79]. Another allogeneic vaccine with a similar structure, viagenpumatucel-L (HS-110), has been obtained transfecting a human lung adenocarcinoma cell line with the fusion gene gp96-Ig. Its combination with nivolumab in NSCLC patients failing an ICI therapy is currently under evaluation on phase I/IIb clinical trial (DURGA trial) [93]. Preliminary results on 20 patients documented a disease control rate of 55%, with 15% ORR. PFS was 2.7 months with a median follow-up of 6 months and there were no grade 5 AEs.

An irradiated autologous tumor cell–based vaccine plus GM-CSF-producing and CD40L-expressing bystander cell line was tested among 24 refractory advanced NSCLC patients in a phase II trial assessing no tumor response [94].

CIMAvax-EGF is a complex vaccine with a peculiar rationale: its composition of P64K, a recombinant Neisseria Meningitis B bacteria–derived carrier protein, conjugated with human recombinant epidermal growth factor (EGF) and Montanide ISA51 as adjuvant, determines the production of anti-EGF antibodies, breaking the immune system tolerance toward EGF and preventing its binding with EGFR [95]. This vaccine proved to be safe and immunogenic in a phase II trials of 80 advanced NSCLC patients who were randomized to BSC or vaccination after completion of first-line chemotherapy [96]. A subsequent phase III trial compared CIMAvax-EGF plus best supportive care (BSC) vs BSC in 405 advanced NSCLC patients after completing first-line chemotherapy [97]. A significant survival benefit was documented, as the median survival time was 12.4 versus 9.4 months in the vaccine and control arm, respectively.

Racotumomab, formerly named 1E10, is a murine anti-idiotype mAb that mimics NeuGcGM3 ganglioside, which is absent in human cytoplasmic membranes but has been detected in several tumors, including NSCLC [98]. Its preliminary efficacy was tested in a compassionate basis study on 34 stage III b and 37 stage IV NSCLC patients after standard chemotherapy and radiotherapy [99]. No serious adverse effects were reported. Median survival was 11.5 months for patients with PS of 0–1 who achieved PR or stable disease (SB) after first-line chemo/radiotherapy, and 6.5 months for those who received the vaccination after progressive disease and/or with a PS of 2. A phase II/III randomized, placebo-controlled trial tested racotumomab as switch maintenance therapy in 176 stage IIIB/IV NSCLC patients who did not progress after first-line chemotherapy [100]. Median OS was 8.2 months in the vaccine group vs 6.8 months in the placebo group and PFS was 5.3 vs 3.9 months in the vaccine and placebo group, respectively. No severe adverse events were reported.

MUC1, a mucin family member, is overexpressed and aberrantly glycosylated in NSCLC [101]. L-BLP25, a liposome vaccine carrying this antigen, fails to demonstrate an OS gain in a phase IIB trial on 171 stage IIB-IV NSCLC not progressing after first-line chemotherapy [102]. The subsequent phase III START trial included unresectable stage III NSCLC which did not progress after completion of chemoradiotherapy, but no benefit in OS was detected [103].

MUC1, together with interleukin 2, was also used to develop TG4010, a cancer vaccine based on a viral vector, a Modified Vaccinia Virus Ankara, encoding for their genes [104]. A phase II trial tested TG4010 with or without first-line chemotherapy in 65 stage IIIB/IV NSCLC patients [105]. OS was 12.7 months in the combination arm versus 14.9 months in the vaccine-only arm. Subsequently, a phase IIb trial assessed the combination of vaccine and chemotherapy versus chemotherapy alone in 148 advanced NSCLC patients expressing MUC1. Six-month PFS, set as the primary endpoint, was 43.2% in the combination group and 35.1% in the chemotherapy alone group. Based on these results, a phase II/III trial enrolled 222 previously untreated stage IV NSCLC patients without EGFR mutation and with MUC1 expression ≥ 50% on tumor cells, to receive standard first-line chemotherapy with or without TG4010 vaccine [106]. The primary endpoint was met, as median PFS was 5.9 months in the TG4010 group and 5.1 months in the placebo group. Other clinical trials evaluating the ongoing studies on vaccines in NSCLC are reported in Table 2.

**Table 2:** Ongoing trials evaluating vaccines safety and efficacy among NSCLC patients

Vaccine	Setting	Phase	Primary outcome	N. Pts ext	clinicaltrial.gov identifier
Viagenpumatucel-L + HS-130	AST refractory to standard care	I	AEs DLT	30	NCT04116710
CIMAvax + nivolumab or pembrolizumab	Advanced NSCLC and HNSCC. Nivolumab arm: after progression on platinum-based chemotherapy Pembrolizumab arm: first-line, PD-L1 > 50%	I/II	DLT OS	181	NCT02955290
Ad/MAGEA3 + MG1-MAGEA3 + pembrolizumab	NSCLC with positive expression of MAGE-A3, progressed after first-line chemotherapy or immunotherapy	I/II	MTD ORR	75	NCT02879760
PDC*lung01 + / − pembrolizumab	Adjuvant in resected stage IIa/IIb/IIIa NSCLC or after 4–6 cycles of platinum-based chemotherapy in stage IV NSCLC	I/II	DLT	66	NCT03970746
Tecemotide (BLP25 liposome vaccine)	Unresectable stage IIIA/IIIB NSCLC	II	AEs	70	NCT00828009
TG4010	First-line, immunotherapy-naive advanced non-squamous NSCLC patients with PD-L1 expression < 50%	II	ORR	39	NCT03353675

*AST* advanced solid tumors, *AEs* adverse events, *DLT* dose-limiting toxicity, *OS* overall survival, *MTD* maximum tolerated dose, *ORR* overall response rate, *NSCLC* non-small cell lung cancer

## Conclusion

PD-1/PD-L1 inhibitors currently occupy a stable spot in the first-line treatment of advanced NSCLC without targetable mutations, which represents the greater quota of NSCLC patients. PD-1/PD-L1 inhibitors in monotherapy and, more recently, in combination with chemotherapy or CTLA-4 inhibitors represent possible choices for the first-line therapy of advanced NSCLC.

**Fig. 3:**
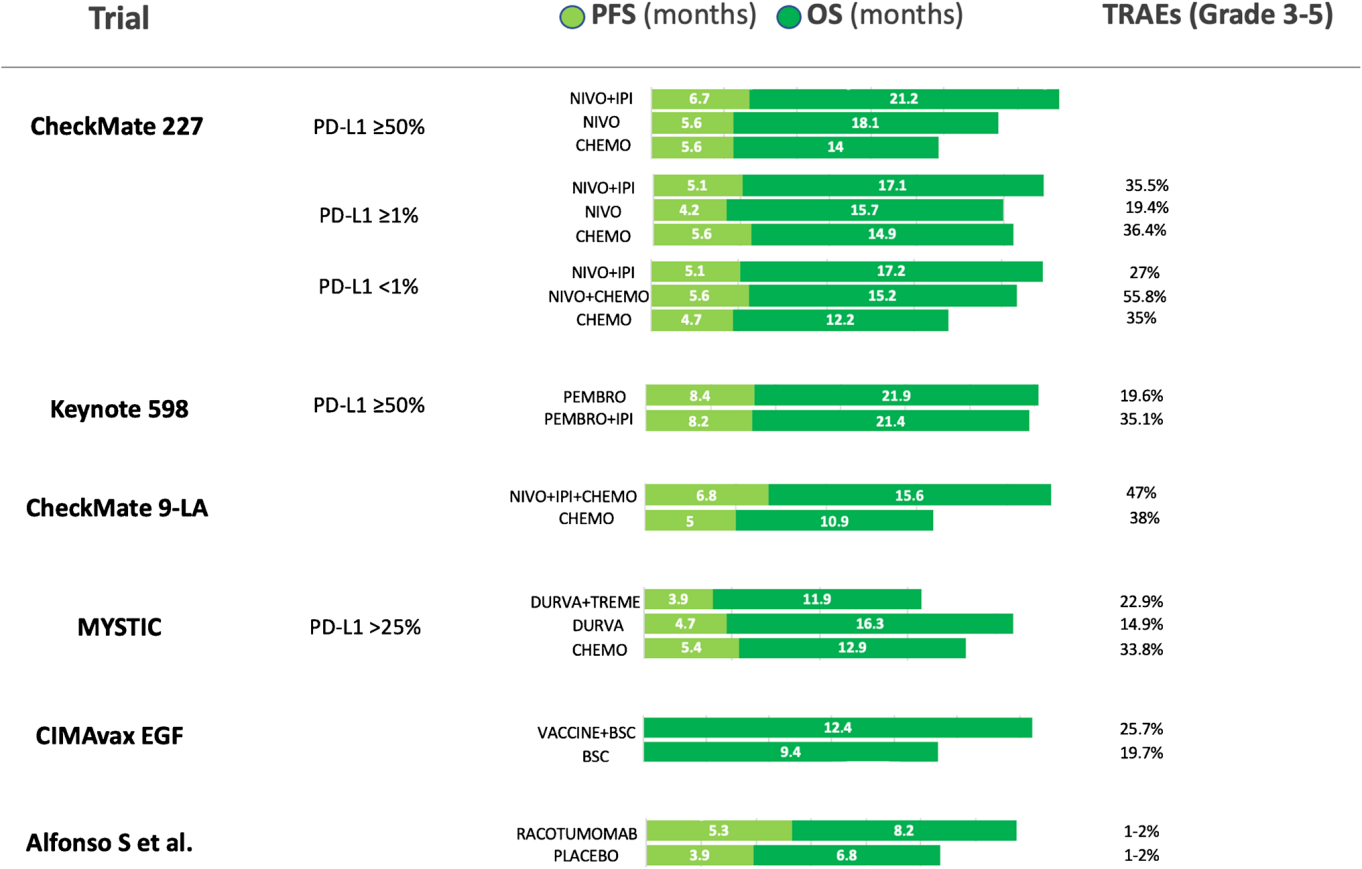
Summary of phase III studies reporting PFS and/or OS data. PFS: progression-free survival; OS: overall survival; TRAEs: treatment-related adverse events; NIVO: nivolumab; IPI: ipilimumab; PEMBRO: pembrolizumab; CHEMO: chemotherapy; DURVA: durvalumab; TREME: tremelimumab

Of all the novel therapeutic agents explored in this review, only trials investigating the use of drugs targeting CTLA-4 showed improved survival outcomes within randomized phase III studies of combination with PD-(L)1 inhibitors (Fig. 3). In particular, the combination of nivolumab and ipilimumab demonstrated OS benefit in first-line, regardless from PD-L1 status and TMB [22]. However, to date, there are no mature data available about a direct comparison with the combination of chemo-immunotherapy, as it recently became the standard of care in the first-line setting. Thus, the role of anti-CTLA-4 after PD-1 inhibition for NSCLC patients is still unclear and further studies are warranted.

In a similar fashion, we believe that the potential of NSCLC vaccines deserves further analysis. Even if the biological rationale underlying the research on cancer vaccines offers interesting perspectives, we witnessed a progressively decreasing interest in this field of research.

Finally, we are looking forward to results from the increasing number of phase II and III clinical trials evaluating the other molecules discussed in this review, with a special focus on TIGIT and OX40 targeting agents, as early reports showed promising results in terms of immune activity and safety.

In conclusion, the landscape of immunotherapy beyond the consolidated role of PD-(L)1 inhibition is wide and offers reliable perspectives of applicability in the next future.
